# MicroRNA-9 regulates survival of chondroblasts and cartilage integrity by targeting protogenin

**DOI:** 10.1186/1478-811X-11-66

**Published:** 2013-09-05

**Authors:** Jinsoo Song, Dongkyun Kim, Churl-Hong Chun, Eun-Jung Jin

**Affiliations:** 1 Department of Biological Sciences, College of Natural Sciences, Wonkwang University, Iksan, Chunbuk 570-749, South Korea; 2 Departments of Orthopedic Surgery, Wonkwang University School of Medicine, Iksan, Chunbuk 570-749, South Korea

**Keywords:** PRTG, miR-9, Apoptotic cell death, Chondrogenesis, Osteoarthritis

## Abstract

**Background:**

Studies have shown the roles of miR-9 and its validated target, protogenin (PRTG) in the differentiation of chondroblasts to chondrocyte and in the pathogenesis of osteoarthritis (OA). We hypothesized that miR-9 plays a distinct role in endochondral ossification and OA pathogenesis and the present study was undertaken to identify this role. In the studies, chondroblasts were isolated from limb bud of chick and mouse embryos and articular chondrocytes were isolated from rabbit and human cartilage. Osteoarthritic chondrocytes were isolated from cartilage from patients undergoing total knee replacement. Using these cells, we analyzed the changes in the expression of genes and proteins, tested the expression level of miR-9, and applied a target validation system. We also performed functional study of miR-9 and PRTG.

**Results:**

With the progression of chondrogenesis, decreased miR-9 level was observed at the time of numerous apoptotic cell deaths. And chondrocytes isolated from normal human articular cartilage expressed miR-9, and this expression was significantly reduced in OA chondrocytes, especially decreased its expression in parallel with the degree of cartilage degradation. Over-expression of PRTG induced the activation of caspase-3 signaling and increased apoptosis. However, the co-treatment with the miR-9 precursor or PRTG-specific siRNA blocked this apoptotic signaling.

**Conclusion:**

This study shows that PRTG is regulated by miR-9, plays an inhibitory action on survival of chondroblasts and articular chondrocytes during chondrogenesis and OA pathogenesis.

## Background

Chondrogenesis is the earliest phase of skeletal development. Most long bones of vertebrates are formed through the process of endochondral ossification. This well-defined and coordinated process involves mesenchymal cell condensation and chondrogenic differentiation for proper cartilage and bone formation [1]. Several reports have shown that two MAPKs, ERK and p38MAPK, regulate chondrogenesis [2,3]. However, despite the importance of these MAPKs in the regulation of cartilage formation, relatively little is known about the involvement of another MAPK signaling pathway, c-jun N-terminal kinase (JNK). Several recent studies demonstrated the importance of JNK signaling during chondrogenesis [4-11]. Activin-A, a member of the transforming growth factor-β family, may suppress chondrocyte differentiation in ATDC5 cells via down-regulation of JNK [4] and reverse signaling of ephrin-B inhibited the attachment and migration of human mesenchymal cells by activating JNK signaling during osteochondral differentiation [5]. Furthermore, in adult articular chondrocytes, MAPK activation is known to associate matrix metalloproteinases (MMPs). Inhibition of JNK signaling inhibits fibronectin fragment stimulation of MMP-13 expression [6,7] and IL-1 stimulation of MMP-13 requires JNK signaling [8]. Our laboratory also showed that JNK signaling is involved in the differentiation of chondroprogenitors in chicks through regulation of miR-34a [9,10] and miR-221 levels [11].

Several reports have suggested a possible role of miRNAs in limb development. In dicer-null mice, a reduced proliferating pool of chondrocytes was observed, and this reduction resulted in severe skeletal growth defects and premature death in the mice [12]. Furthermore, expression of several miRs, including miR-10b and miR-196, was detected in the developing limb and found to be involved in the specification of limb development [13,14]. However, the precise roles of miRNAs in limb development have not yet been fully established.

Protogenin (PRTG) belongs to the immunoglobulin superfamily and is most closely related to the deleted in colorectal cancer (DCC)-Neogenin subclass [15], which, in addition to DCC and Neogenin, includes Punc and Nope. Recent study showed that PRTG have two proteolytic cleavages. One is between the fibronectin III and the transmembrane domain for ectodomain-shedding, another is by γ-secretase at the interface of the transmembrane and the intracellular domain to release C-terminal intracellular domain of PRTG. This released C-terminal intracellular domain can translocate to the nucleus to regulate neuronal differentiation [16]. PRTG functions as a receptor to prevent precocious neuronal differentiation in neural progenitors [17] and plays a role in the rearrangement of cells of the paraxial mesodermal lineage [18]. Recently, the expression pattern of PRTG in mouse embryos has been published [19]. As in mouse embryos, PRTG became progressively restricted dorsally in the spinal cord with highest level in the roof plate anterior to the forelimb, suggesting a role during avian limb development. Although several studies emphasize the importance of PRTG during development of various tissues, neither a specific role nor the molecular mechanisms of PRTG action during limb development have been determined. The factors responsible for PRTG regulation are also still unknown. Here, for the first time, we found that PRTG exhibits chondro-inhibitory action in limb mesenchymal cells and that PRTG is a direct target of miR-9.

## Results

### MiR-9 induces chondro-inhibitory action during chondrogenic differentiation of chick limb mesenchymal cells

**Figure 1:**
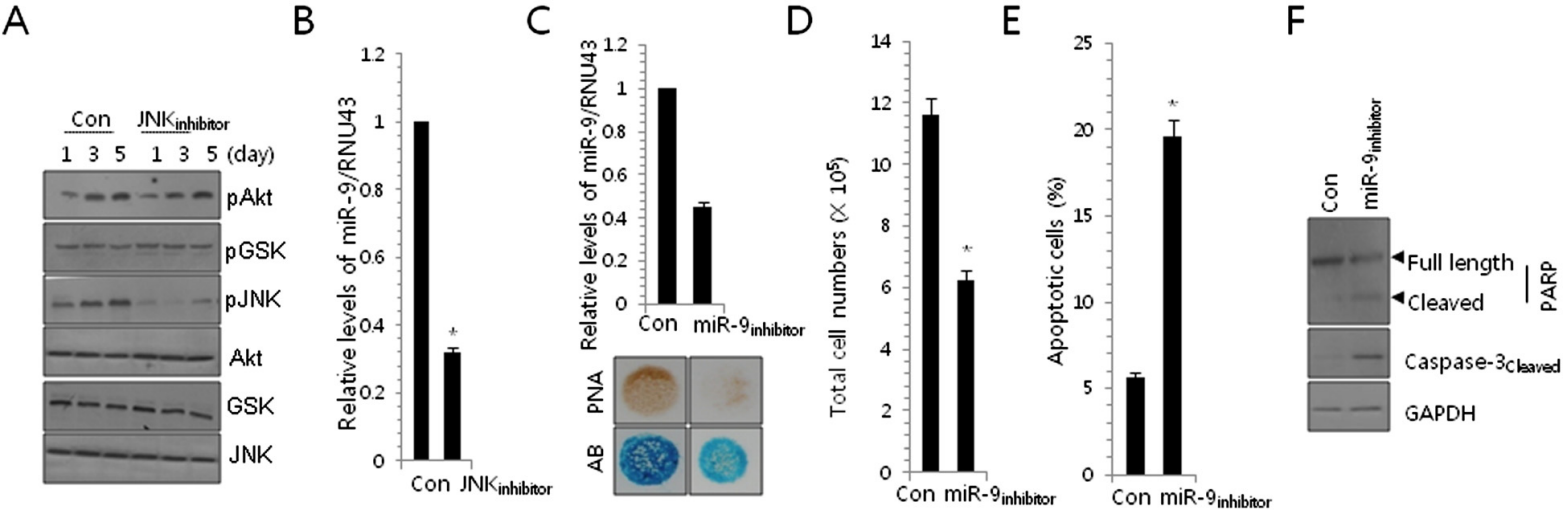
**MiR-9 affects cell proliferation and survival during chondrogenesis of chick limb mesenchymal cells. (A)** Changes in the phosphorylation levels of Akt, GSK, and JNK were analyzed by Western blotting. **(B)** Total RNA was purified from chondroprogenitors cultured with or without 5 μM JNK inhibitor and the expression of mir-9 was measured with real-time PCR. **(C)** Chondroprogenitor cells were treated with 100 nM of anti-mir-9 oligonucleotides (mir-9inhibitor). The expression of mir-9 was measured with real-time PCR (upper panel) and Precartilage condensation was analyzed by PA staining at day 3 and Alcian blue staining at day 5 of culture (lower panel). The data shown are representative of at least four independent experiments. The diameter of typical standard culture is 5 mm. **(D)** Total cell numbers were counted at 2 day of culture. **(E)** Apoptotic cells were analyzed by FACS analysis. **(F)** Changes in the cleaved form of caspase-3 were analyzed by Western blotting. Results of cell adhesion experiments were pooled from 5 replicate samples derived from 4 independent experiments. The mean is plotted and the error bars represent 95% CI (lower/upper limit). ***, statistically different from control cells (p < 0.001).

From previously reported miRNA array data by inhibition of JNK signaling [11], we identified 14 up-regulated miRNAs and 12 down-regulated miRNAs whose expressions were altered during chondrogenesis (Additional file 1). Among them, miR-9 was one of miRNA whose expression was substantially altered with inhibition of chondrogenic differentiation (determined using a P-value of 0.01 as a cutoff for significance). Inhibition of JNK signaling did not affect other signaling, including Akt and GSK, as confirmed by immunoblotting (Figure 1A). Down-regulation of miR-9 by blockade of JNK signaling was confirmed by quantitative RT-PCR (Figure 1B).

In order to examine the involvement of miR-9 during chondrogenesis, we exposed mesenchymal cells to 200 nM peptide nucleic acid-based antisense oligonucleotides (ASOs) against miR-9 (miR-9 inhibitor) whose knockdown efficiency was monitored by real time PCR (Figure 1C, upper panel). Precartilage condensation and chondrogenic differentiation were assessed by PA at day 3 and Alcian blue staining at day 5, respectively. Decreased intensities of PA at day 3 and Alcian blue staining at day 5 were observed with treatment of anti-miR-9 oligonucleotides (Figure 1C, lower panel). Treatment of cells with a miR-9 inhibitor caused a significant decrease in total cell numbers (Figure 1D) with significant increases in apoptotic cell death (Figure 1E) and caspase-3 activity (Figure 1F). Our results revealed that miR-9 inhibitor-induced apoptotic cell death may be responsible for JNK blockade-induced chondro-inhibitory action on precartilage condensation.

### MiR-9 stimulated chondrogenic differentiation by regulating protogenin

**Figure 2:**
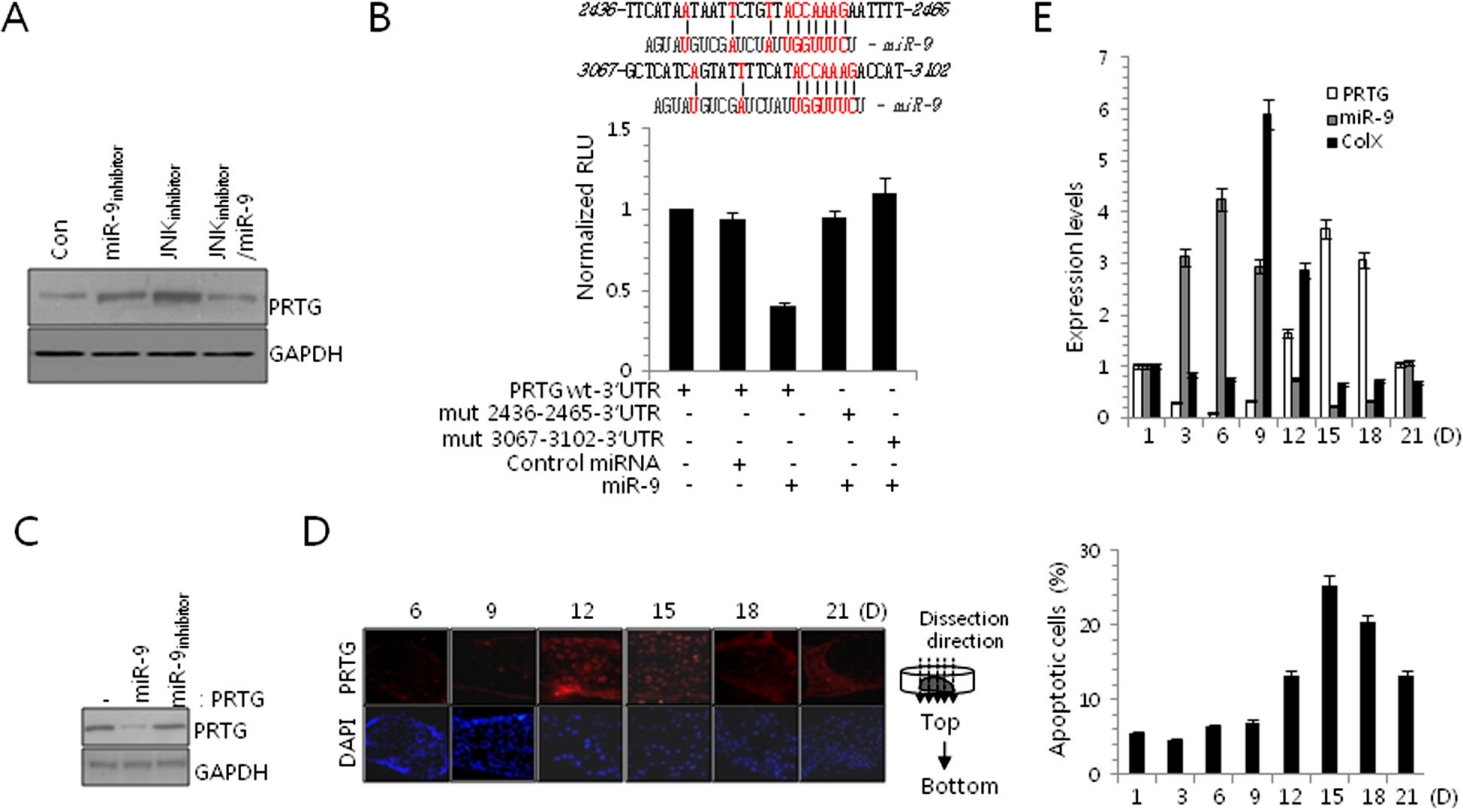
**miR-9 targets PRTG and inhibits chondrogenic differentiation. (A)** Cells were cultured at a density of 2 × 10^7^ cells/ml and treated with miR-inhibitor, JNK inhibitor or in the combination of JNK inhibitor and miR-9 precursor (miR-9). Changes in the protein level of PRTG were analyzed at day 2 of culture. **(B)** Luciferase reporter gene assays of cells expressing the construct containing the human PRTG-3′-UTR or mutated seed sequence (mut 2436–2465, mut3067-3102) of putative targets (upper panel) in the absence or presence of miR-9. **(C)** Cells were electroporated with PRTG, incubated with either miR-9 precursor or miR-9 inhibitor, and changes in the protein level of PRTG were analyzed at day 2 of culture. **(D)** Mouse chondrogenitors were cultured at a density of 2 × 10^7^ cells/ml, sectioned, and stained with anti-PRTG antibody at day 6, 9, 12, 15, 18, 21 days of culture. **(E)** The expressions of PRTG, type X collagen (Col X), and miR-9 were measured with real-time PCR. Apoptotic cells were analyzed by FACS analysis. One-way analysis of variance (ANOVA) with Tukey post hoc comparisons of groups was used to test for significant effects. The mean is plotted and the error bars represent 95% CI (lower/upper limit). ***, statistically different from control cells (p < 0.001).

Target genes of miR-9 were predicted using miRNA target prediction algorithms, including TargetScan and miRDB and *PRTG* was identified as a potential target. In support of this prediction, we observed a significant induction in PRTG protein level in miR-9 inhibitor-treated or JNK inhibitor-treated chondroprogenitor cells. And increased protein level of PRTG by JNK inhibitor treatment was significantly reduced with co-introduction of miR-9 (Figure 2A). To confirm that PRTG is a target for miR-9, we cloned the entire 3′ UTR of PRTG into a luciferase reporter vector, electroporated the vector into chondrogenic progenitors along with the precursor of miR-9 or a cognate non-targeting negative control, and assayed cell lysates for luciferase expression. We found that cells transfected with the PRTG-3′ UTR vector plus miR-9 exhibited significantly less luciferase activity compared to cells that received the vector plus the non-targeting negative control (Figure 2B). Seed sequences of putative targets for miR-9 (Figure 2B upper panel) were exchanged a purine for a pyrimidine and a pyrimidine to a purine. Luciferease activity was not affected with these mutated constructs. Induction of miR-9 successfully reduced PRTG protein level in myc-tagged *PRTG*/pCAGGS vector electroporated cells (Figure 2C).

To investigate temporal and spatial expression of PRTG, micromass cultures were sectioned longitudinally and immunostained with PRTG antibody (Figure 2E). The RNA level of PRTG was also significantly decreased at 3, 6, and 9 days of culture i.e. at the time of proliferation and condensation with increased expression level of miR-9 and significantly increased at 12, 15, and 18 days of culture, i.e. at the time of hypertrophy and apoptosis with a decreased expression level of miR-9 (Figure 2F).

### MiR-9 protects PRTG-induced apoptosis of chondroprogenitors during chondrogenesis

**Figure 3:**
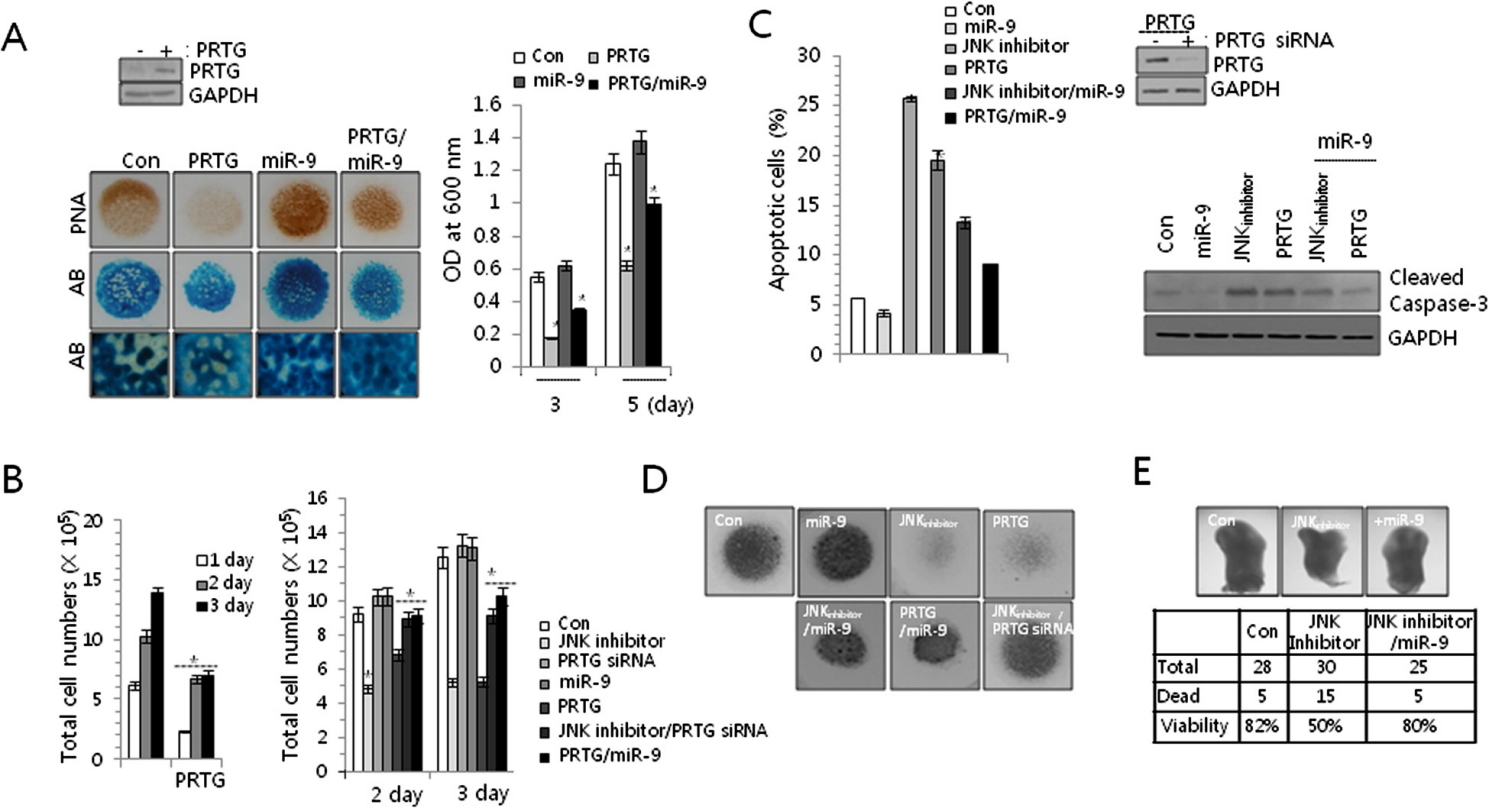
**PRTG induced apoptotic death of chondroprogenitors. (A)** Cells were electroporated with *PRTG*/pCAGGS (PRTG) construct in the absence or presence of miR-9 precursor (miR-9) and electroporation efficiency was confirmed by immunoblotting (left upper panel). Precartilage condensation and chondrogenic differentiation were analyzed by PA staining at day 3 and Alcian blue staining at day 5 of culture, respectively (left lower panel) and chondrogenesis was quantified by measuring the absorbance of bound Alcian blue at 600 nm at day3 and day5 of culture (right panel). **(B)** Cells were electroporated with PRTG construct and the number of viable cells was determined at 1, 2, and 3 day of culture (left panel), treated with JNK inhibitor or miR-9, electroporated with PRTG or PRTG siRNA, or in the combination of JNK inhibitor and PRTG siRNA or PRTG and miR-9 and the number of viable cells were determined at day 2 and 3 of culture (right panel). **(C)** Apoptotic cells were analyzed by FACS analysis (left panel) and changes in the cleaved form of caspase-3 were analyzed by Western blotting (right panel). **(D)** Cells were treated with JNK inhibitor in the combination of miR-9 or PRTG-specific siRNA, or introduced with miR-9 in the combination of PRTG. Precartilage condensation and chondrogenic differentiation were analyzed by PA staining at day 3. The diameter of typical standard culture is 5 mm. **(E)** HH stage 18 chick embryos (wing bud) were treated with JNK inhibitor in the presence or absence of miR-9 precursor and incubated for additional 2 days (HH stage23). The number of embryos used for each experiment is represented as a table (upper panel), and a representative image of each limb is shown (lower panel). The mean is plotted and the error bars represent 95% CI (lower/upper limit). *, statistically different from control cells (p < 0.001).

To observe the effects of PRTG, chondroblasts were electroporated with the myc-tagged *PRTG*/pCAGGS vector and the transfection efficiency was confirmed by immunoblotting (Figure 3A left upper panel). Precartilage condensation markedly decreases in response to PRTG over-expression (Figure 3A left lower panel). When the micromass cultures were stained with Alcian blue, the number and size of individual cartilage nodules (Figure 3A left lower panel) and staining intensities (Figure 3A right panel) were also noticeably decreased in response to PRTG over-expression. And these inhibitory actions of PRTG on precartilage condensation and chondrogenic differentiation were recovered by co-introduction of miR-9. These data suggested that miR-9 suppresses sulfated proteoglycan accumulation and cartilage nodule formation for chondrogenic differentiation possibly by targeting PRTG.

Since condensation could be due to the modulation of cell number, we next examined whether PRTG suppresses precartilage condensation and chondrogenic differentiation through regulation of cell proliferation or survival. Consistent with suppression of chondrogenesis, cell proliferation was significantly decreased in PRTG over-expressed cells (Figure 3B left panel). Furthermore, decreased in total cell number by JNK inhibitor or PRTG was reversed by co-introduction of PRTG siRNA or miR-9, respectively (Figure 3B, right panel). Apoptotic cell death, as assessed by FACS analysis (left panel) and by caspase-3 activity (right panel), was increased by the introduction of PRTG or treatment of JNK inhibitor and inhibited by co-induction of miR-9 (Figure 3C). As well, inhibited precartilage condensation by JNK inhibition and PRTG over-expression was recovered by co-electroporation of PRTG-specific siRNA or co-introduction of miR-9 (Figure 3D) confirmed its efficiency with PRTG over-expressed cells (Figure 3C lower panel).

To further investigate miR-9 involvement in limb formation, 18 HH stage chick embryos were treated with JNK inhibitor in the absence or presence of miR-9 inhibitors. We observed the disruption of limb formation, especially formation of inter-digital regions, in JNK inhibitor-treated chick embryos. This malformation was overcome by co-treatment of miR-9 inhibitor (Figure 3E). These results indicate that negative regulation of chondrogenesis by the over-expression of PRTG is mediated by modulating apoptotic death of chondrogenic competent cells.

### MiR-9 also protects PRTG-induced apoptosis of chondrocytes

**Figure 4:**
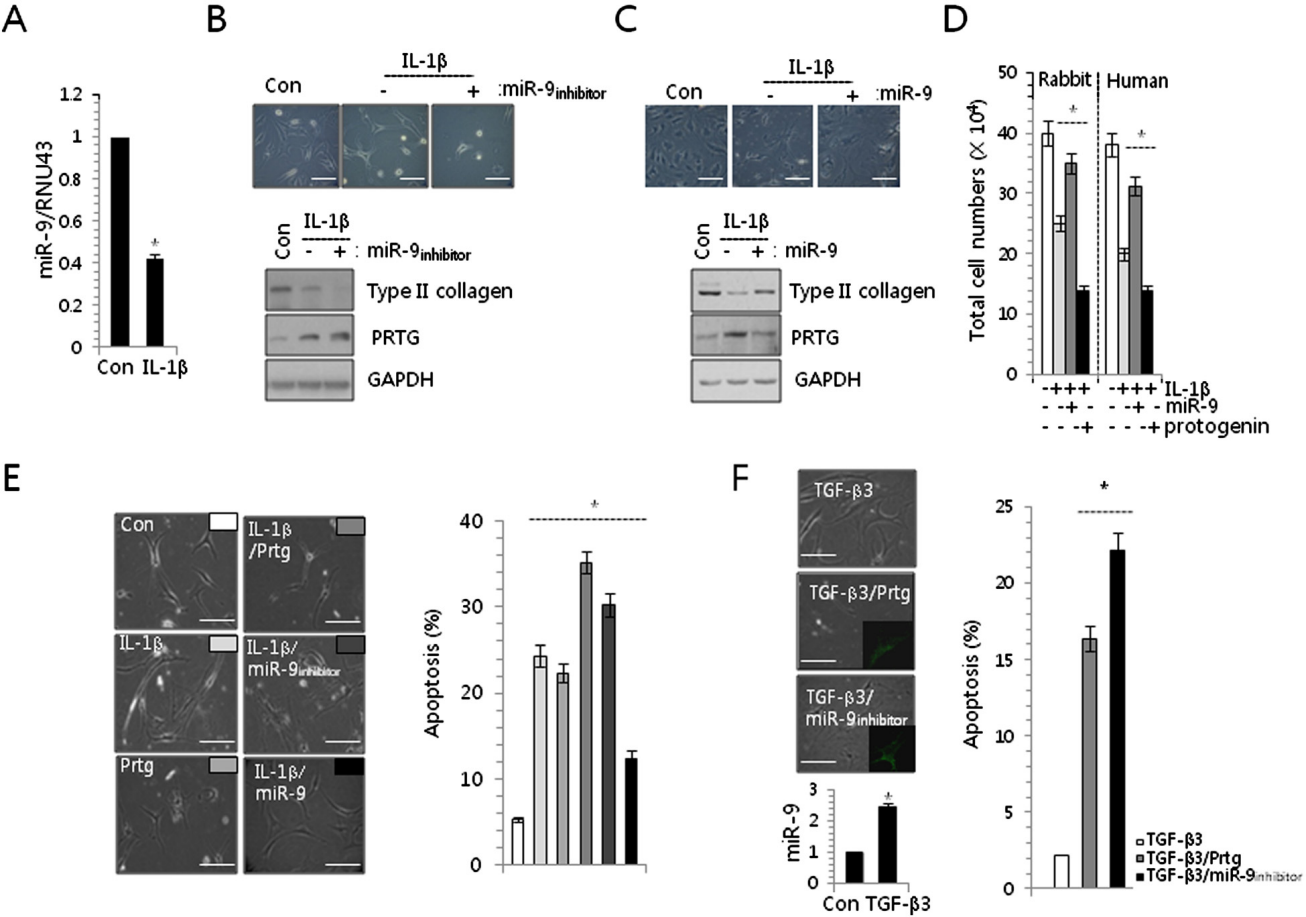
**miR-9 is also involved in the degeneration of articular chondrocytes. (A)** Rabbit articular chondrocytes were treated with 5 nM IL-1β in the absence or presence of 100 nM of the miR-9 inhibitor. Change in expression level of miR-9 in was analyzed by real-time PCR. **(B)** Images of the cultures were captured using light microscopy (Upper panel). Changes in the protein level of Type II collagen and PRTG during chondrogenesis were analyzed by Western blotting (Lower panel). GAPDH was used as control. **(C)** Rabbit articular chondrocytes were treated with 5 nM IL-1β in the absence or presence of miR-9 precursor. Images of the cultures were captured using light microscopy (Upper panel). Changes in the protein level of Type II collagen and PRTG during chondrogenesis were analyzed by Western blotting (Lower panel). GAPDH was used as control. **(D)** Rabbit and human articular chondrocytes were treated with 5 nM IL-1β in the presence of 100 nM of the miR-9 inhibitor or the PRTG construct. The number of viable cells was determined at 2 day of culture. **(E)** Human articular chondrocytes were electroporated with PRTG, miR-9 inhibitor, or miR-9 in the absence or presence of IL-1β (left panel) and apoptotic cell death (right panel) was analyzed. **(F)** Human articular chondrocytes isolated from biopsy normal cartilage were electroporated with Prtg or miR-9 inhibitor in the presence of TGF-β3 and apoptotic cell death was analyzed. Change in expression level of miR-9 in was analyzed by real-time PCR. *, statistically different from control cells (p < 0.001). The error bars represent average of data from each human sample. Scale bar, 200 μm.

In order to further study the role of miR-9 in survival of chondrocytes, dedifferentiation of articular chondrocytes was induced by IL-1β exposure. We confirmed that IL-1β exposure to cells decreased the expression level of miR-9 (Figure 4A). It has been shown that differentiated chondrocytes could lose their intrinsic characteristics upon exposure to IL-1β [20,21], nitric oxide [22], or retinoic acid [23,24], and during serial monolayer culture [25,26] through a process designated “dedifferentiation”. Dedifferentiation was confirmed by a degenerated morphology (Figure 4B and 4C upper panel). A more significant degenerative phenotype and decreased level of type II collagen were observed in co-treatment of miR-9 inhibitor with IL-1β (Figure 4B) and IL-1β-induced degenerative changes were prevented by co-introduction of miR-9 (Figure 4C). Consisted with these observations, the protein level of PRTG was increased by co-treatment of miR-9 inhibitor (Figure 4B) and decreased by co-introduction of miR-9 (Figure 4C). The total cell number of rabbit articular chondrocytes and human articular chondrocytes was decreased with IL-1β treatment. A more significant decrease was observed with co-treatment of miR-9 or PRTG (Figure 4D).

**Figure 5:**
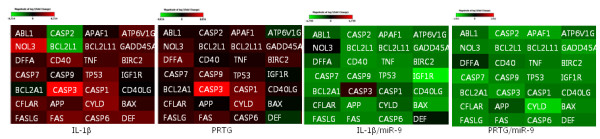
**miR-9 and its target, PRTG is involved in chondrocyte apoptosis.** Human articular chondrocytes isolated from biopsy normal cartilage were electroporated with Prtg or miR-9 in the absence or presence of IL-1β and expression levels of apoptotic genes were examined and represented as heat-map.

For further investigation of involvement of miR-9 or PRTG, macroscopically normal human cartilage from 10 adult donors from both genders (mean age 37.4 years; age range 20–60 years), without history of joint disease was confirmed that the specimens were histological normal cartilage and used for isolating primary articular chondrocytes. A significant degenerative phenotype was observed with IL-1β-treated or PRTG-introduced chondrocytes (Figure 4E left panel). Most significant degeneration was observed in the combination of IL-1β and PRTG-treated cell or in the combination of IL-1β and miR-9 inhibitor-treated cell. However, IL-1β-induced degeneration was significantly blocked by co-introduction of miR-9. We also observed that increased apoptotic cell death by IL-1β was blocked by co-introduction of miR-9 (Figure 4E right panel). In addition, co-introduction of PRTG or inhibition of miR-9 significantly increased apoptosis in cells treated with TGF-β3 (Figure 4F), a known positive regulator of chondrocytes [27]. For further validation for apoptotic involvement of miR-9 and PRTG, normal chondrocytes were introduced with miR-9 in the absence or presence of IL-1β or PRTG and expression levels of genes involved in apoptosis was examined (Figure 5). Apoptotic genes including ABL1, ATP6V1GNOL3, CASP1, 3, 7, CD40, CYLD, and FAS were induced with IL-1β treatments or PRTG over-expression whereas expression levels of those genes were decreased with miR-9 introduction.

### MiR-9 also involves in the pathogenesis of osteoarthritis

**Figure 6:**
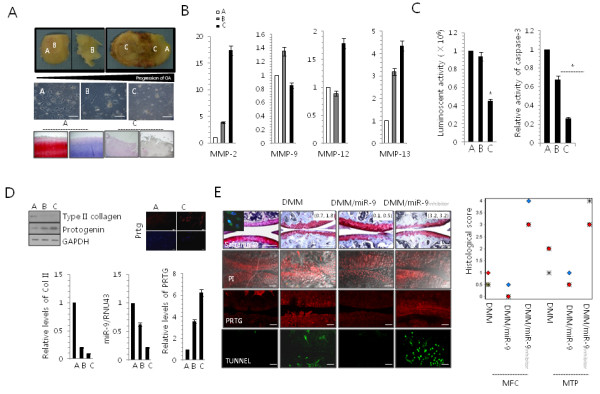
**MiR-9 is involved in pathogenesis of OA.** Articular chondrocytes were isolated form cartilage (upper panel) that was divided into 3 classes depending on the progression of OA pathology (A: healthy zone, B: intermediate zone, and C: severe zone). **(A)** Images of the cultures were captured using light microscopy and human cartilages were stained with safranin O and Alcian blue. **(B)** The expressions of MMP-2, MMP-9, MMP-12, and MMP-13 were measured with real-time PCR. **(C)** Cell viability (right panel) and caspase-3/7 activity (left panel) were analyzed. **(D)** Changes in the protein level of Type II collagen and PRTG were analyzed by Western blotting. GAPDH was used as control (upper left panel). PRTG expression was analyzed by immunocytochemisty (upper right panel). The expressions of type II collagen (Col II), PRTG, and miR-9 were analyzed by real-time PCR (lower panel). **(E)** Mouse cartilages with OA induced by destabilization of the medial meniscus (DMM), were infected with miR-9 or si-miR-9 lentiviruses and stained with safranin O, propium iodide, and Tunnel. PRTG level was analyzed by immunohistochemistry (left panel). Inserted number in safranin-O photomicrographs indicated the averages of semi-quantitative score for the degree of cartilage destruction in MFC (first score) and MTP (second score) view. Each histological score for the degree of cartilage destruction (n = 5 mice/group) in MFC (first score) and MTP (second score) view were graphed (right panel). Sham-operated (Sham) cartilage was used as control. *, statistically different from control cells (p < 0.001). The error bars represent average of data from each human sample. Scale bar, 200 μm.

To investigate the pathological involvement of miR-9, 10 osteoarthritic (OA) cartilage was obtained from patients diagnosed with OA according to the American College of Rheumatology (ACR) criteria, which underwent joint surgery (mean age 64.6 years; age range 52–71 years). Knee radiographs from the OA participants were classified as grade IV according to the Kellgren and Lawrence (K/L) scoring system (Figure 6A). OA cartilage was divided into non-OA region (A), mild-OA region (B), and severe-OA region (C, Figure 6A upper panel) as confirmed by a degenerative morphology with OA progression (Figure 6A middle panel) and staining with Safranin O and Alcian blue (Figure 6A lower panel).

Proteolytic degradation of cartilage is a hallmark of OA and activated chondrocytes are known to produce matrix-degrading enzymes such as collagenase 3 (MMP-13) in OA joints [28]. Expression of MMP-13 in mice resulted in pathologic changes in the joints, similar to human OA [28]. In addition, the proinflammatory cytokine interleukin-1 (IL-1β) and MMP-13 localize to the site of cartilage degradation in OA joints, providing evidence of their key roles in the pathogenesis of OA [29,30]. Consistent with previous reports [28,30], the expression levels of MMP-2, −12, and −13 (Figure 6B) were increased. Furthermore, cell viability was significantly decreased in area C and the caspase-3 activity was significantly increased in area B and C (Figure 6C). The protein and RNA levels of type II collagen and miR-9 were decreased whereas those levels of PRTG were increased as the progression of cartilage damage (Figure 6D).

To validate the role of miR-9 in chondrocyte apoptosis during OA cartilage destruction *in vivo*, we overexpressed miR-9 in cartilage tissue by injecting miR-9-expressing or si-miR-9 expressing lentiviruses into DMM mouse knee joints (Figure 6E). Cartilage destruction as visualized by safranin-O staining was significantly induced by DMM surgery. Semi-quantitative scoring for cartilage destruction [31] using safranin-O photomicrographs of medial femoral condyle (MFC) and medial tibial plateau (MTP) indicated that DMM surgery scored as 0.5 by MFC view and 2 by MTP view. Most severe cartilage destruction was observed with the infection of si-miR-9 expression lentiviruses (MFC score of 3, MTP score of 3). However, over-expression of miR-9 significantly reduced cartilage destruction (MFC score of 0, MTP score of 0.5). Consistent with this, increased apoptosis of articular chondrocytes and PRTG level by DMM surgery was also inhibited with over-expression of miR-9 and stimulated with suppression of miR-9.

## Discussion

During development, most of our bones form through endochondral ossification in which bones are first laid down as cartilage precursor [1] and mitogen-activated protein kinase (MAPK) cascades are known to play essential roles in regulating mesenchymal cell chondrogenesis [2,3]. Particularly, our recent study showed the involvement of JNK signaling during chondrogenesis of limb mesenchymal cells [11]. We reported the involvement of several miRNAs including miR-34a [9,10] and miR-221 [11] in JNK-regulated chondrogenic differentiation. Here, we found another miRNA, miR-9 involved in JNK-induced chondrogenic differentiation. Furthermore, we suggested that miR-9 is one of important players in OA pathogenesis.

MiRNAs play key roles in diverse regulatory pathways, including cell proliferation, differentiation, apoptosis, and many other physiological and pathological processes [32,33]. However, the precise roles of miRNAs in cartilage biology are largely unknown. Here, we investigated the functional importance of miR-9 both in endochondral ossification and OA pathogenesis.

MiR-9 provides a model for controlling the balance between neural stem cell proliferation and differentiation [34]. MiR-9 is known as a growth inhibition factor and plays a role as in anti-proliferative activity in human gastric adenocarcinoma cells by negatively targeting NF-κB1 at the post-transcriptional level [35]. Jones and colleagues (2009) suggest the involvement of miR-9 in OA bone and cartilage by mediating the IL-1β-induced production of TNF-α [36]. Here, we show that miR-9 targets PRTG, thus revealing a potential mechanism for apoptotic death of limb chondroblasts during endochondral ossification. Experimental evidence indicates that PRTG is a target of miR-9. First, the ability of miR-9 to regulate PRTG expression is likely direct, because it binds to the 3′UTR of PRTG mRNA. Second, the luciferase intensity of PRTG-UTR was specifically responsive to miR-9 over-expression suggesting that miR-9 may regulate PRTG protein expression by inducing translational suppression. Consistent with the results obtained with PRTG over-expression, knock-down of miR-9 promoted the apoptotic death of limb chondroblasts. Our study provides evidence for the mechanism through which miR-9 affects the survival/proliferation of chondrocytes and PRTG is one of the physiologic targets of miR-9 in the regulation of chondrocyte survival.

In this study, we also sought to determine the effect of PRTG in chondrogenic differentiation and the regulatory mechanism of PRTG, a member of the immunoglobulin superfamily that is most closely related to DCC-Neogenin subclass [37]. The ability of Neogenin to regulate cell death appears to be dependent on the context of its expression, i.e. certain cell types respond differently to cell death signaling. Over-expression of Neogenin in chick dorsal root ganglion neurons has no noticeable effect on cell survival [37], whereas in PC12 cells, Neogenin induces apoptosis [38]. Knockdown of Neogenin in zebrafish increased apoptotic cell death and reduces neuronal differentiation [39]. Our results revealed for the first time that PRTG exerts chondro-inhibitory effects through up-regulation of apoptotic cell death on limb chondroblasts.

Here, we also suggest the involvement of miR-9 in OA pathogenesis as well as chondrogenic differentiation of limb mesenchymal cells. OA is a progressive degenerative disease characterized by cartilage degradation and chondrocyte apoptosis. In addition, chondrocyte apoptosis in osteoarthritic cartilage has been reported in dogs, humans, and horses [40,41] and is considered to be one of the major factors in the pathogenesis of the OA disease process. Here, we also found that cell viability was decreased in degenerated rabbit and human articular chondrocytes and miR-9: PRTG interplay is involved in the apoptotic process of IL-1β-induced degeneration. It has been shown that miR-9 is responsible for regulating viability of chondrocytes and reduction of miR-9 was observed in generative chondrocytes and this could be a reason for decreasing cell viability.

The primary pathogenic events in OA include loss and abnormal remodeling of cartilage extracellular matrix. Chondrocytes are the major cell type of the articular cartilage and function to maintain tissue homeostasis. Recent findings indicate that chondrocyte death and survival are closely linked with cartilage matrix integrity [42]. Two key targets of cartilage degeneration during OA are type II collagen and aggrecan [43]. The accumulation of degraded fragments over time increase MMP-13 synthesis and leads to positive feedback loop through interaction with cell-surface integrins resulting destruction of knee joints [44]. Yang and collegues (1997) found increased chondrocyte apoptosis in transgenic mice lacking type II collagen [45]. Our laboratory (2010) also showed that degradation of type I collagen by MMP-9 stimulated cell death, by interfering with cell attachment and integrin-mediated survival signaling [46]. These previous reports suggest that degradation of cartilage matrix could be an inducer for chondrocyte apoptosis. However, it still remains unclear whether chondrocyte apoptosis is a cause of, or the result of, cartilage matrix breakdown. Cells require attachment to the extracellular matrix (ECM) for survival, function, and growth. A disruption of the collagen network could disturb chondrocyte anchorage to the ECM and result in chondrocyte apoptosis. Alternatively, cartilage homeostasis could not be maintained due to chondrocytes apoptosis, and therefore cartilage degradation could be induced.

We observed an increased protein level of MMP-13, a major cartilage degrading enzyme, with increasing stages of OA pathogenesis. In OA, a progressive degenerative disease, proteolytic degradation of cartilage by matrix-degrading enzymes, such as MMP-13 [47,48] and ADAMTS5 [49,50], is a hallmark. MiR-146a functions in an anti-catabolic manner in articular cartilage by antagonizing the IL-1β-induced expression of cartilage-degrading enzymes MMP13 [51] and ADAMTS5 [52]. Reduced miR-140 expression was observed in human OA cartilage [53,54]. MiR-140 plays dual roles in both cartilage development and homeostasis, in part via by regulating Adamts-5 in OA [55]. Our laboratory is currently undergoing study on the relationships between miR-9, PRTG, and MMP-13 to verify whether chondrocyte apoptosis by PRTG, a target for miR-9, is down-stream, up-stream, or independent of MMP-13 induction.

In sum, here, for the first time, we found that PRTG is regulated by miR-9, resulting in an inhibition of cell proliferation and survival in chondrogenic progenitors and articular chondrocytes. Reduction of miR-9 induction, which results in increased PRTG levels in OA pathogenesis, may be responsible for chondrocyte apoptosis, a typical hallmark of OA.

## Methods

### Primary cell cultures

Mesenchymal cells (at a density of 2 × 10^7^ cells/ml) were derived from the distal tips of Hamburger-Hamilton (HH) stage 22/23 embryo limb buds of fertilized White Leghorn chicken eggs or E11.5 embryos. They were micromass cultured in Ham’s F-12 medium containing 10% fetal bovine serum (FBS), 100 IU/ml penicillin, and 100 μg/ml streptomycin (Gibco Invitrogen, Grand Island, NY). A concentration of 5 μM was chosen for JNK inhibitor II (Calbiochem, San Diego, CA) and treated for entire culture period in this study.

Rabbit articular chondrocytes from joint cartilage slices of 2-week-old New Zealand white rabbits were isolated with 0.2% collagenase type II, as described previously [56] and were then plated on culture dishes at a density of 5 × 10^4^ cells/cm^2^. The medium was replaced every 2 days after seeding.

Human articular cartilage specimens were obtained from cartilages that were undergoing total knee arthroplasty. Tissue collection was approved by the Human Subjects Committee of Wonkwang University. Chondrocytes were extracted as previously described [57] and seeded at a density of 1.5 × 10^4^ cells/cm^2^ in DMEM (Gibco-Invitrogen) supplemented with 10% fetal bovine serum (FBS), 100 units/ml penicillin, and 100 μg/ml streptomycin (Gibco Invitrogen). A concentration of 5 ng/ml was chosen for IL-1β (R & D systems, Minneapolis, MN) in this study.

### Analysis of cell differentiation and precartilage condensation

Alcian blue bound to sulfated glycosaminoglycans was extracted with 6 M guanidine-HCl, and quantified by measuring the absorbance of the extracts at 600 nm. Cultures were incubated with 100 μg/ml biotinylated peanut agglutinin (PA, Sigma) and visualized with the VECTASTAIN ABC and DAB substrate solution kit (Vector laboratories Inc., Burlingame, CA).

### Apoptosis assay

Apoptosis was analyzed by a flow cytometer (FACS calibure, Becton-Dickinson, France). To detect extent of propidium iodide, cells were excited at 488 nm and emission was observed at 585 nm.

### Caspase assay

Activities of caspase-3 and caspase-7 were determined using a caspase colorimetric assay kit (R&D Systems Inc., Minneapolis, MN, USA).

### Cell viability assay

Cell viability was assayed using CellTiter-Glo luminescent cell viability assay kit (Promega), which determines viability based on the quantification of ATP present in metabolically active or viable cells [58].

### Cell proliferation assay

Proliferation was determined by direct counting of cells. Control and treated cultures were detached with trypsin/EDTA solution and counted in triplicate using a hematocytometer.

### Western blot analysis

Total proteins (30 μg) were electrophoresed and transferred to nitrocellulose membranes (Schleicher and Schuell, Keene, Germany). The membranes were individually probed with antibodies specific for Type I, II collagen, PRTG (Calbiochem, La Jolla, CA), (p)AKT, (p)GSK, (p)JNK, GAPDH (Santa Cruz Biotechnology Inc.), Caspase-3, PARP (Cell Signaling Technology Inc., Danvers, MA, USA). The blots were developed using a peroxidase-conjugated secondary antibody, and the immunoreactive proteins were visualized with an ECL system (Amersham, UK).

### Electroporation

Chondrogenic progenitors were electroporated with either a myc-tagged PRTG (PRTG) expression vector (a kind gift from Dr. D. Watanabe at Department of Molecular Neurobiology, Institute of Development, Aging and Cancer, Tohoku University, Japan; pCAGGS was used as mock) or PRTG-specific siRNA (purchased from Invitrogen, PRTG_stealth primers; 5′-UUUACAGGUAAAUCGAGCUACUCCA-3′, 5′-UGGAGU AGCUCGAUUUACCUGUAAAA-3′) using a BTX-830 square wave generator (Gentronics, San Diego, CA) with 20 msec, 200 square pulses.

### MiRNA and mRNA real-time quantitative RT-PCR

MiRNA and mRNA expression were independently quantified using the TaqMan MicroRNA and TaqMan gene expression assays (Applied Biosystems), respectively, according to the manufacturer’s protocols. MiRNA expression was normalized to RNU43 small nuclear RNA endogenous controls.

For mRNA, transcripts were quantified by real-time quantitative polymerase chain reaction (RT-PCR) and normalized to the amount of GAPDH mRNA expressed The oligonucleotides used as primers were listed in Table 1.

**Table 1:** The list of primers

**Mouse gene**	**Primers**	
PRTG	5′-aagtcaatgacgggcatcgcagta-3′	5′-acttcctggcttgcttcggtaga-3′
Type X collagen	5′-ataagaacggcacgcctaagatgt-3′	5′-ctgcattgggcattggagccata-3′
GAPDH	5′-tgtccgtcgtggatctgac-3′	5′-cctgcttcaccaccttcttg-3′
**Human gene**	**Primers**	
type II collagen	5′-tcactcatgccctgaag-3′	5′-ctatgtccatgggtgcaatg-3′
PRTG	5′-tgcatgcaagat tcatcccaccc-3′	5′-tgcaatactcctgttggtagggca-3′
MMP-2	5′acaccaagaacttc gtctg-3′	5′-tgcagatctcaggagtgaca-3′
MMP-9,	5′-atttctgccaggaccgcttctact-3′	5′-atgtcataggtcacgtagcccact-3′
MMP-12	5′-gaaccaacgcttgccaaatcctga-3′	5′-ttcccacggtagtgacagcatcaa-3′
MMP-13	5′-ttgcagagcgctacctgagatcat-3′	5′-tttgccagtcacctctaagccgaa-3′
ABL1	5′-gaagcccaaaccaaaaatgg-3′	5′-gactgttgactggcgtgatgtag-3′
AFAF1	5′-tgcgctgctctgccttct-3′	5′-gcggagcacacaaatgaaga-3′
APP	5′-tgtccgcgc agaacagaa-3′	5′-tgtccgcgcagaacagaa-3′
ATP6V1G2	5′-ggaaaacatcctgacttcagtgtct-3′	5′-ccagcaagtgacagggtcaa-3′
BAX	5′-ccaaggtgccgg aactga-3′	5′-cccggaggaagtccaatgt-3′
BCL2A1	5′-cctggatcaggtccaagcaa-3′	5′-ttggactgagaacgcaacattt-3′
BCL2L11	5′-gctttcccatggtcacaggat-3′	5′-ctgcagctggactctgctgta-3′
BIRC2	5′-cctgtggtgggaagctcagt-3′	5′-cctccg gtgttctgacatagc-3′
CASP1	5′-at accaagaactgcccaagtttg-3′	5′-ggcaggcctggatgatga-3′
CASP2	5′-ggtaaagaaaagttgccgaagatc-3′	5′-ggcatag ccgcatatcatgtc-3′
CASP3	5′-gcc tacagcccatttctccat-3′	5′-gcgccctggcagcat-3′
CASP6	5′-ggcgtggttactcacacctgta-3′	5′-gatccgcccaccttgga-3′
CASP7	5′-ccgccgtgggaacgat-3′	5′-cctcaaccccctgctcttc-3′
CASP9	5′-agcagtgggctcactctgaag-3′	5′-aacagcattagcgaccctaagc-3′
CD40	5′-tggtgagtgactgcacagagttc-3′	5′-cgctttcaccgcaagga-3′
CD40LG	5′-ccaggtgcttcggtgtttgt-3′	5′-ccagtgccatggctcactt-3′
CFLAR	5′-gctggcagctgattagatggt-3′	5′-tttgagtcagtggactgggaaa-3′
CYLD	5′-tgtggagggcttgcaatgt-3′	5′-agctgagatgtcc ggatcgt-3′
DEFB1	5′-ttgacgctccctgctcaga-3′	5′-tggacggtggcacaactct-3′
FAS	5′-acccgctcagtacggagttg-3′	5′-ccagcatggttgtt gagcaa-3′
FASLG	5′-tgcctcctcttgagcagtca-3′	5′-tcctgtagaggctgaggtgtca-3′
GADD45A	5′-gatgtggctctgcagatcca-3′	5′-atgtcgttctcgc agcaaaa-3′
IGF1R	5′-cttgtacattcgca ccaatgct-3′	5′-cgattaactgagaagaggagttcga-3′
NOL3	5′-gcccaccacgagcatca-3′	5′-cctggactcctaag ggcagat-3′
TNF	5′-gcccaccacgagcatca-3′	5′-cctggactcctaag ggcagat-3′
TP53	5′-tgcaataggtgtgcgtcagaa-3′	5′-ccccg ggacaaagcaaa-3′
GAPDH	5′-gatcatcagcaatgcctcct-3′	5′-tgtggtcatgagtccttcca-3′

*ABL1* c-abl oncogene1, non receptor tyrosine kinase, *AFAF1* apoptotic peptidase activating factor1, *APP* amyloid beta-A4 pre cursor protein, *ATP6V1G2* ATPase, H + transporting lysosomal 13 kDa, V1 subunit G2, *BAX* BCL2-associated × protein, *BCL2A1* BCL2-related protein A1, *BCL2L1* BCL2-like 1, *BCL2L11* BCL2-like 11, B, *IRC2* baculoviral IAP repeat containing 2, *CASP1* caspase 1- apoptosis-related cysteine peptidase, *CASP3* caspase 3- apoptosis-related cysteine peptidase, *CASP9* caspase 9- apoptosis-related cysteine peptidase, *CD40* CD40 molecule, TNF receptor super family member 5, *CD40LG* CD40 ligand, *CFLAR* CASP8 and FADD-like apoptosis regulator, *CYLD* cylindromatosis-turban tumor syndrome, *DEFB1* defensin, beta 1, *DFFA* DNA fragmentation factor, 45 kDa, alpha polypeptide, *FAS* Fas-TNF receptor super family, member 6, *FASLG* Fas ligand-TNF super family, member 6, *GADD45A* growth arrest and DNA-damage-inclucible, alpha, *IGF1R* insulin-like growth factor 1 receptor, *NOL3* necleolar protein 3-apoptosis repressor with CARD domain, *TNF* tumor necrosis factor, *TP53* tumor protein p53.

### Synthesis of a PNA (peptide nucleic acid)-based miRNA inhibitor and induction in cells

PNA, an artificially created DNA analogue, exhibits superior binding affinity and chemical/biological stability because the phosphate ribose ring of DNA is replaced with a polyamide backbone. The PNA-based ASOs, which contain an O-linker at the N terminus of the PNA to improve solubility, were purchased from Panagene (Korea). A scrambled PNA-based ASO was used as a negative control (5′-RRRQRRKKR-00-ATTAATGT CGGACAA-3′, RRRQRRKKR: cell penetrating peptide; O:AEEA linker) and 200 nM of PNA-based ASO (PNA9: UCUUUGGUUAUCUAGCUGUAUGA) were electroporated into isolated mesenchymal cells.

### Reporter vectors and DNA constructs

The 3′-UTR of human PRTG (PRTG) was PCR amplified using the following primers: 5′-TGGGAGCTCCTGGCTCTATT-3′ (bp no. 1616 ~ 1635), 5′-GCTGAGGCTGACTTT GCACT-3′ (bp no. 3088 ~ 3107). It was then cloned downstream of the CMV-driven firefly luciferase cassette in the pMIR-REPORT vector (Ambion). For miRNA target validation, chondroblasts were electroporated with 200 ng of a firefly luciferase reporter construct, 50 pmol of pre-miR-9 or pre-miR-negative (Ambion). The Renilla luciferase vector was used to normalize electroporation efficiency. At 24 hr after electroporation, both firefly and Renilla luciferase activity were assayed (Promega). Normalized relative light units represent firefly luciferase activity or Renillar luciferase activity.

### Arthritic cartilage, experimental OA, and histology of OA cartilage

International Cartilage Repair Society (ICRS) grade 10 human OA cartilage was sourced from individuals (age 51–72 years) undergoing arthroplasty for OA of the knee joint. The Wonkwang University Hospital Institutional Review Board approved the use of these materials, and all individuals provided written informed consent before the operative procedure. Human OA cartilage samples were frozen, sectioned at a thickness of 10 μm, fixed in paraformaldehyde, and stained with Alcian blue.

Experimental OA was induced by destabilization of the medial meniscus (DMM) surgery 8-week-old male mice. Sham-operated animals injected with empty lentiviruses (mock transduction) were used as controls for DMM. Mice were killed 8 weeks after DMM surgery or 2 weeks after intraarticular injection (1 × 10^9^ plaque-forming units (PFU)) of miR-9-expressing lentiviruses (lenti-miR-9) for histological and biochemical analyses. Cartilage destruction in mice was examined using Safranin O staining. Briefly, knee joints were fixed in 4% paraformaldehyde, decalcified in 0.5 M EDTA (pH 7.4) for 14 days at 4°C, and embedded in paraffin. The paraffin blocks were sectioned at 6 μm thickness. The sections were deparaffinized in xylene, hydrated with graded ethanol, and stained with Safranin O.

### Tunnel assay

Apoptosis of articular chondrocytes in cartilage tissues was determined by TUNEL assay using a kit from Clontech (Mountain View, CA). Specimens were visualized under a fluorescence microscope.

### Immunohistochemistry

Deparaffinized section was incubated with the anti-PRTG antibody (1: 200 dilutions) overnight at 4°C, followed by incubation with rhodamine-conjugated secondary antibody at room temperature for 1 hour. Specimens were visualized under a fluorescence microscope.

### Statistical analysis

Statistical analysis was performed using the SPSS program for Windows, Standard Version (version 18.0, SPSS Inc., Chicago, http://www.SPSS.com).

## Competing interests

The authors declare that they have no competing interests.

## Authors’ contributions

All authors were involved in drafting the article critically for important intellectual content. EJJ and CHC had full access to all of the data in the study and take responsibility for the integrity of the data and the accuracy of the data analysis. Study conception and design. JS, ML, DK, CHC, EJJ Acquisition of data. JS, DK, Analysis and interpretation of data. ML, CHC, EJJ. All authors read and approved the final manuscript.

## Supplementary Material

Additional file 1 Differentially expressed miRNAs at 48 hr after suppression of JNK signaling in limb mesenchymal cells. Click here for file
