# Correction to: MicroRNA-9 regulates survival of chondroblasts and cartilage integrity by targeting protogenin

**DOI:** 10.1186/s12964-019-0483-7

**Published:** 2019-11-28

**Authors:** Jinsoo Song, Dongkyun Kim, Churl-Hong Chun, Eun-Jung Jin

**Affiliations:** 1 Department of Biological Sciences, College of Natural Sciences, Wonkwang University, Iksan, Chunbuk 570-749 South Korea; 2 Departments of Orthopedic Surgery, Wonkwang University School of Medicine, Iksan, Chunbuk 570-749 South Korea

**Correction to: Cell Commun Signal (2013) 11:66**

**http://www.biosignaling.com/content/11/1/66**

Following publication of the original article [1], the authors reported that Figs. 3 and 6 are incorrect.

There are errors in a Safranin O staining image for DMM/miR-9 in Fig. 6e and Alcian blue staining images for control and miR-9 in Fig. 3a. The correct figures are supplied below.

**Figure 1:**
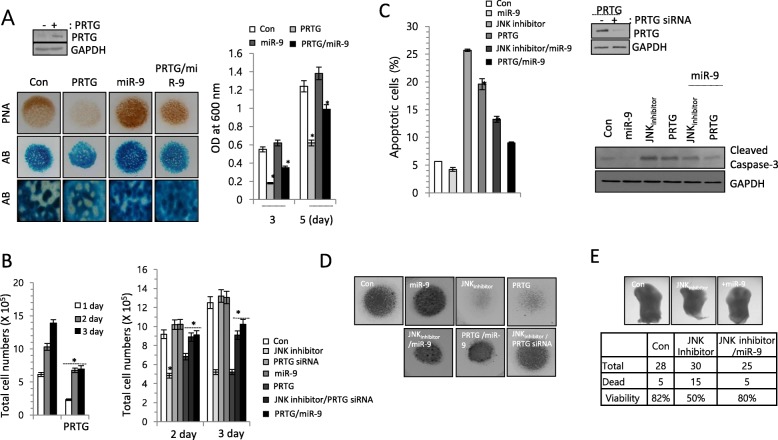
**Fig. 3**

**Figure 2:**
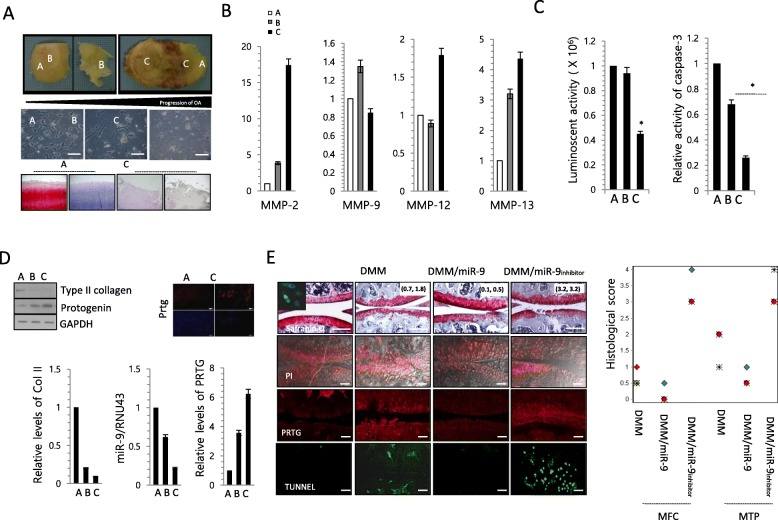
**Fig. 6**
