# Supplementary material for: MicroRNA-9 regulates survival of chondroblasts and cartilage integrity by targeting protogenin
Source: Cell Commun Signal. 2013 Sep 5;11:66. doi: 10.1186/1478-811X-11-66 (PMC3848287; doi:10.1186/1478-811X-11-66)
Supplement: Additional file 1 — Differentially expressed miRNAs at 48 hr after suppression of JNK signaling in limb mesenchymal cells. [file 1478-811X-11-66-S1.ppt]

## Slide 1
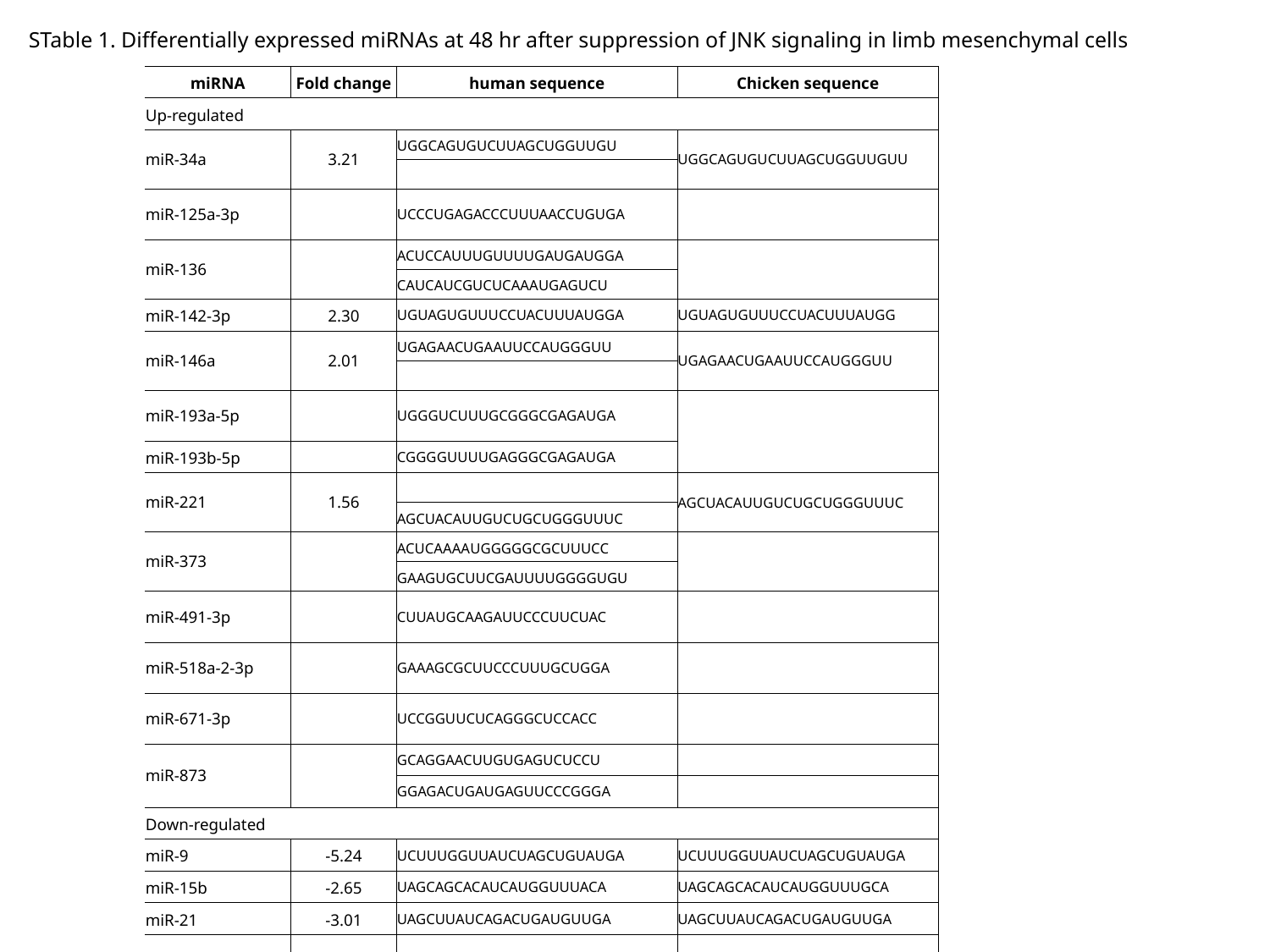

STable 1. Differentially expressed miRNAs at 48 hr after suppression of JNK signaling in limb mesenchymal cells
| miRNA | Fold change | human sequence | Chicken sequence |
| --- | --- | --- | --- |
| Up-regulated | | | |
| miR-34a | 3.21 | UGGCAGUGUCUUAGCUGGUUGU | UGGCAGUGUCUUAGCUGGUUGUU |
| | | | |
| miR-125a-3p | | UCCCUGAGACCCUUUAACCUGUGA | |
| miR-136 | | ACUCCAUUUGUUUUGAUGAUGGA | |
| | | CAUCAUCGUCUCAAAUGAGUCU | |
| miR-142-3p | 2.30 | UGUAGUGUUUCCUACUUUAUGGA | UGUAGUGUUUCCUACUUUAUGG |
| miR-146a | 2.01 | UGAGAACUGAAUUCCAUGGGUU | UGAGAACUGAAUUCCAUGGGUU |
| | | | |
| miR-193a-5p | | UGGGUCUUUGCGGGCGAGAUGA | |
| miR-193b-5p | | CGGGGUUUUGAGGGCGAGAUGA | |
| miR-221 | 1.56 | | AGCUACAUUGUCUGCUGGGUUUC |
| | | AGCUACAUUGUCUGCUGGGUUUC | |
| miR-373 | | ACUCAAAAUGGGGGCGCUUUCC | |
| | | GAAGUGCUUCGAUUUUGGGGUGU | |
| miR-491-3p | | CUUAUGCAAGAUUCCCUUCUAC | |
| miR-518a-2-3p | | GAAAGCGCUUCCCUUUGCUGGA | |
| miR-671-3p | | UCCGGUUCUCAGGGCUCCACC | |
| miR-873 | | GCAGGAACUUGUGAGUCUCCU | |
| | | GGAGACUGAUGAGUUCCCGGGA | |
| Down-regulated | | | |
| miR-9 | -5.24 | UCUUUGGUUAUCUAGCUGUAUGA | UCUUUGGUUAUCUAGCUGUAUGA |
| miR-15b | -2.65 | UAGCAGCACAUCAUGGUUUACA | UAGCAGCACAUCAUGGUUUGCA |
| miR-21 | -3.01 | UAGCUUAUCAGACUGAUGUUGA | UAGCUUAUCAGACUGAUGUUGA |
| miR-122 | | UGGAGUGUGACAAUGGUGUUUG | UGGAGUGUGACAAUGGUGUUUGU |
| miR-124-1 | | CGUGUUCACAGCGGACCUUGAU | |
| | | UAAGGCACGCGGUGAAUGCC | |
| miR-140 | -3.56 | CAGUGGUUUUACCCUAUGGUAG | AGUGGUUUUACCCUAUGGUAG |
| miR-136 | | ACUCCAUUUGUUUUGAUGAUGGA | |
| | | CAUCAUCGUCUCAAAUGAGUCU | |
| miR-199b | -2.98 | ACAGUAGUCUGCACAUUGGUUA | CAGUAGUCUGCACAUUUGGU |
| miR-367 | | AAUUGCACUUUAGCAAUGGUGA | AAUUGCACUUUAGCAAUGGUG |
| miR-488 | -3.45 | CCCAGAUAAUGGCACUCUCAA | |
| | | UUGAAAGGCUAUUUCUUGGUC | |
| miR-551b | -3.22 | GCGACCCAUACUUGGUUUCAG | GCGACCCAUACUUGGUUUCAG |
| miR-566 | | GGGCGCCUGUGAUCCCAAC | |
| miR-571 | | UGAGUUGGCCAUCUGAGUGAG | |
| miR-622 | | ACAGUCUGCUGAGGUUGGAGC | |
| miR-632 | | GUGUCUGCUUCCUGUGGGA | |
